# Social and economic barriers to adherence among patients at Livingstone General Hospital in Zambia

**DOI:** 10.4102/phcfm.v11i1.1740

**Published:** 2019-04-16

**Authors:** Kaala Moomba, Brian van Wyk

**Affiliations:** 1 School of Public Health, Faculty of Community and Health Sciences, University of the Western Cape, Bellville, South Africa

**Keywords:** adherence, HIV, treatment, barriers, social, economic

## Abstract

**Background:**

Zambia is one of the countries hardest hit by the human immunodeficiency virus (HIV) and acquired immune deficiency syndrome (AIDS) pandemic with a national HIV prevalence estimated at 14% among those aged 15–49 years in 2012. Antiretroviral therapy (ART) has been available in public health facilities in Zambia since 2003. By early 2016, 65% of the 1.2 million Zambians living with HIV were accessing ART. While access to ART has improved the lives of people living with HIV globally, the lack of adherence to ART is a major challenge to treatment success globally.

**Aim:**

This article reports on social and economic barriers to ART adherence among HIV patients being attended to at Livingstone General Hospital in Zambia.

**Setting:**

Livingstone General Hospital is located in the Southern province of Zambia, and had over 7000 patients enrolled for HIV care of whom 3880 patients were on ART.

**Methods:**

An explorative, qualitative study was conducted with 42 patients on ART where data were collected through six focus group discussions (3 male and 3 female groups) and seven in-depth interviews. Data were audio-recorded and transcribed verbatim and subjected to thematic content analysis.

**Results:**

Economic factors such as poverty and unemployment and the lack of food were reported as major barriers to adherence. Furthermore, social factors such as traditional medicine, religion, lack of family and partner support, and disclosure were also reported as critical barriers to adherence to ART.

**Conclusion:**

Interventions to improve adherence among ART patients should aim to redress the socio-economic challenges at community and individual levels.

## Introduction

Zambia has one of the largest human immunodeficiency virus (HIV) epidemics in sub-Saharan Africa, with a HIV prevalence of 14.3% in 2012 among the 15–49-year-old population.^1^ Of the estimated 36.9 million people living with HIV and/or acquired immune deficiency syndrome (AIDS) (PLWHA) globally by the end of 2014, Zambia accounted for approximately 1.2 million cases, with about 55 000 new infections among children and adults occurring in that same year.^2^ In 2002, the Zambian government made a commitment towards fighting the AIDS epidemic by adopting the National AIDS Strategic Framework, which provides an overall strategy for the planning, coordination and implementation of the multi-sectoral national response.^2^ As of early 2016, over 70% of PLWHA eligible for antiretroviral therapy (ART) had been initiated on treatment in the country.^2^

Antiretroviral therapy has been proven to significantly reduce the viral loads of PLWHA and forms an integral part of the comprehensive approach used in curbing the AIDS epidemic.^3^ While the availability of highly active antiretroviral therapy (HAART) improves the lives of PLWHA, sub-optimal adherence to ART among patients remains a major challenge and can lead to the development of resistant strains and ultimately to regimen failure.^3,4^ In order to inhibit viral activity, above 95% adherence to antiretroviral (ARV) drugs is required.^5^

Several factors contributing to poor adherence to ART have been identified in both developed and developing countries.^6,7^ Numerous studies have shown that economic factors such as transport costs and lack of food interfere with adherence.^8,9^ Social factors such as traditional and cultural beliefs, stigma and discrimination, religion and family support, partner support and disclosure (or lack of disclosure) can also negatively influence ART adherence.^10,11,12,13,14,15,16^ Regimen characteristics such as side effects, pill burden and the complexity of treatment have also been shown to significantly affect adherence to ART.^11,12,17,18^ Patient-related characteristics such as (younger) age and (male) gender, alcohol and other drug use, low literacy or educational levels, mental health, patients’ forgetfulness and daily schedules have also been shown to negatively influence adherence.^10,11,19,20,21,22,23,24^ Health service factors have also been reported to affect adherence negatively. Negative staff attitudes towards HIV patients, inadequate counselling, lack of confidentiality, long waiting times at clinics, long travel distances to ART clinics, poor health information, poor patient–health provider relations, inadequate skills and staff competencies are all health services barriers that have been reported to negatively impact on patient adherence to ART.^21,23,25,26^ Although the above-mentioned factors that influence adherence are known in general terms, it is imperative that the factors (drivers) influencing poor adherence in the local settings are identified in order to develop targeted interventions.

## Research methods and design

### Problem

The Health Management Information System (HMIS) at Livingstone General Hospital (LGH) in Zambia shows poor adherence among patients accessing HAART. The ART statistics for the 6-month period from January to June 2011 showed nearly 10% (343 out of 3880) of patients having missed their monthly refills by 2–30 days – implying adherence to medication of < 95%. In addition, a review of the hospital data showed that there has been an increase in the number of patients that have been changed from the first-line regimen to the second-line therapy because of treatment failure, which is usually because of poor patient adherence to ART. It is for this reason that this study was designed to explore the factors that hinder adherence among patients on ART at LGH.

### Study design

An explorative qualitative study approach was used. According to Sankar et al., the qualitative approach is well suited for gathering in-depth information about human experiences, which helps us understand the reasons for certain behavioural patterns.^27^ The use of a qualitative approach was motivated by the fact that the researcher was interested in getting an in-depth understanding of issues surrounding patients’ adherence or non-adherence to ART at LGH, based on their experiences and perspectives.

### Study population and sampling

The study population comprised adult patients attending the ART clinic and accessing ART at LGH. In this study, purposive sampling was employed to ensure maximum variation among the study sample. The inclusion criteria included treatment status and gender. Both male and female patients who were treatment naïve (starters less than 6 months on ART), experienced (more than 1 year but less than 3 years on ART) and long term (more than 3 years on ART), with varying experience, were included in the sample. A total of 42 male and female patients participated in the study. A two-stage recruitment and consent process was followed. The researcher worked closely with a registry clerk working at the ART clinic to recruit participants for the study. The clerk was trained on how to identify patients that meet the inclusion criteria (age above 18 years, months or years on ART and gender). The patients were identified and approached by the clerk during their regular clinic visit and the study was explained. After a brief explanation about the study, the patients were asked if their contact details could be forwarded to the researcher. If patients agreed, they were requested to fill in a ‘consent to be contacted by the researcher’ form with their details. Whenever a patient declined or indicated no interest, the next eligible patient was approached. The contact details were forwarded to the researcher and the second stage of recruitment was done by the researcher who contacted the participants and made formal arrangements for the focus group discussions (FGDs) and the individual semi-structured interviews.

The demographic characteristics of the participants are summarised in Table 1.

**TABLE 1:** Demographic characteristics of participants (*N* = 42).

Variable	*N*
**Sex**	
Male	23
Female	19
**Age (in years)**				
20–29	4
30–39	14
40–49	19
50–59	4
60+	1
**Marital status**	
Married	32
Single	10
**Years on ART**		
6–12 months (treatment naïve)	7
Between 1 and 3 years (experienced)	12
> 3 years (long term)	23

### Data collection

Data were collected by the researcher through six FGDs (3 female and 3 male groups) and follow-up in-depth interviews with seven of the focus group participants. Each FGD comprised 6–10 participants. An FGD guide and a semi-structured interview guide, which were grounded in the literature, were used. Studies conducted in similar settings were reviewed to inform the guides used in this study with open-ended questions used to probe on the subject under study to facilitate further discussion. Both tools were used throughout the study to ensure standardisation and consistency across the FGDs and interviews. The FGDs and in-depth interviews were conducted in the language of the participants’ choice (English, Tonga or Nyanja) to enable them to express themselves fully. These were conducted in a suitable private conference room where the respondents could talk freely. The FGDs and semi-structured interview guides comprised open-ended questions which explored various aspects of the patient’s experiences of adherence to ART. Participants for the semi-structured interviews were recruited from among members of the FGDs who showed considerable knowledge on the subject under study. Upon reaching data saturation, no further FGDs and semi-structured interviews were conducted. The FGD and semi-structured interviews were digitally recorded and transcribed verbatim. Member checking was done during and after FGDs and interviews to determine accuracy of the information. Confidentiality was ensured by using coded names for the participants with their true identities kept secret, which strengthened trustworthiness.

### Data analysis

Prior to data analysis, transcriptions were translated in to English, where necessary. The data were analysed manually using the thematic content analysis. This was guided by the five stages of thematic analysis, namely familiarisation stage, theme identification stage, indexing stage, charting stage, and the mapping and interpretation stages.^28^ Using these stages, themes and sub-themes were identified and consensus reached after which a hierarchy of themes was constructed. The sub-themes obtained were presented under economic factors, social factors, patient characteristics and health services factors.

### Ethical considerations

Ethics approval was obtained from the Biomedical Research Ethics Committee of the University of the Western Cape (registration number 11/9/26). Further approval was obtained from the Provincial Medical Office and Livingstone General Hospital. Participation in the study was voluntary, and participants were free to withdraw from the study without being penalised in any way. A participant information sheet with an explanation of the purpose and nature of the study was provided to the participants and explained in their language of choice. They were asked to sign a consent form before participating in the study. Anonymity in the study was ensured by keeping all data under lock and key throughout the study.

## Results

The most salient themes that influenced treatment adherence were categorised as *economic factors* and *social factors*. The themes and sub-themes are presented in Table 2 and described below, with supporting quotes from participants.

**TABLE 2:** Themes and sub-themes reported to influence adherence to antiretroviral therapy.

Themes	Sub-themes
Economic factors	Poverty and unemployment
	Lack of food
Social factors	Traditional and cultural beliefs
	Religion
	Family support, partner support and disclosure

### Economic factors

Economic factors such as poverty, unemployment and lack of food were consistently mentioned by participants in relation to the question about their experiences on ART.

#### Poverty and unemployment

Most participants were unemployed and experienced poverty as a result of this. The lack of income meant that most of them were unable to buy food or afford transportation costs to meet their ART clinic appointment dates:

‘Like me … I’m not working. It is hard to meet the demands from home. [Putting] food on the table is hard. Sometimes you just eat breakfast; no lunch and supper or supper but no lunch. It’s hard.’ (Participant 2, male, 34 years)

‘I stay far away so I always have to look for money to come. I stay in Kazungula which is about 70 km away from here. It’s a problem to come here.’ (Focus group 6, female, 42 years)

Some participants stay very far from the health facilities and are thus challenged to make follow-up clinic appointments, especially during the rainy seasons:

‘Here you find there are people living in the outskirts of the district like Sinde and Jack Mwanapapa. Those places are far from here. During the rainy season, there are streams which are filled with water. Someone would want come but for them to travel it is difficult. So, they come after a week or two.’ (Participant 7, male, 45 years)

Even among those participants who were employed, the nature of their employment at times affected their ability to adhere to the drugs, as they often have to travel. The nature of some jobs was also such that their schedules were not regular to support adherence:

‘… I’m a journalist. You are out for weeks, months. For you to travel back to pick up drugs - you cannot and your due date finds you in another place.’ (Participant 2, male, 34 years)

‘Like he has said. I’m a businessman when time comes you are out. So you fail to collect your drugs.’ (Focus group 3, male, 31 years)

‘There is a young man who got employed as a casual worker and when he went he did not take enough drugs. He did not have drugs for about two months and he came back shaking and staggering. He had to be restarted on the drugs.’ (Focus group 1, male, 50 years)

The participants also reported that they often simply forgot to take their medication at the prescribed times because they were busy at (with) work or in their homes:

‘Sometimes because of my work I have forgotten to take my medication but not the whole day maybe 30 minutes but as soon as I remember, I take it. Even at church where I am also a secretary to the pastor, when there are conferences running late I tend to forget but I am getting used to taking them now.’ (Participant 5, female, 37 years)

‘As ladies, what affects us a lot is homework; you are sweeping or cooking by the time you remember, it’s past your time.’ (Focus group 2, female, 28 years)

#### Lack of food

Most of the participants reported that they often suffer hunger or do not have enough food to eat to ensure that their stomachs are sufficiently lined so that they are able to take the ARV drugs without severe side effects:

‘If food is little, these drugs give appetite, they are powerful. If you are not strong you can stop taking them.’ (Participant 2, male, 34 years)

‘Hunger is the biggest problem. Many don’t work and the medication we take is very strong. It would be good if it can be looked into then things will be better for others.’ (Focus group 3, male, 41 years)

‘You have no food; so people are fearing to take these drugs. I don’t know what you can do. I don’t work … If we would be given food that would help. That’s why a lot of people stop drinking their medicines.’ (Focus group 5, male, 32 years)

### Social factors

Social factors such as traditional medicine, religion, and family support, partner support and disclosure of HIV status were identified by participants as hindrances to adherence to ART.

#### Use of traditional medicine

The use of traditional medicine was a common practice in the setting. Although none of the participants reported (themselves) replacing ART with traditional medicine, they did report that acquaintances of theirs replaced ART drugs with traditional medicines. One male participant, for example – who has been on ART for between 1 and 3 years – reported how he combined his ART medication with traditional medicine:

‘Yes, so far so good. You take it with tea or any foodstuff. In food, you put one teaspoon. It has worked. Immediately I started using it the CD4 improved.’ (Focus group 1, male, 36 years)

‘I had a friend who did not come for three months for ARV collection, when we met, he told me he was taking something else.’ (Focus group 5, male, 32 years)

‘Here people are saying there is ‘Back to Eden’ (herbs) so I have a friend who is refusing to take ARVs even his children. He is promoting herbal things. When I looked at his children, he now has mental problem because of using Back to Eden.’ (Focus group 3, male, 41 years)

#### Religion

Religion played a big role in the research setting. Some participants reported that certain religions promoted faith healing and how such people (followers of the religion) were encouraged to abandoned ART when the priests told them to have faith that they were healed through prayer. There were mixed reports about some HIV patients being ‘healed’ by faith and others becoming sick again or even dying:

‘Concerning religion, so many have died because of being cheated by pastors telling them just to have faith and they will be okay. I have seen two or three die because they believed pastors and stopped taking their drugs.’ (Participant 3, male, 45 years)

‘This issue we are talking about ART - one woman is sick right now. My neighbour, she went to church and was told she was healed and threw away her ARVs. She came back and is not healed. She got very sick and now she is back on ART.’ (Focus group 3, male, 31 years)

‘If you take ARVs and you want to be prayed for, then you have no faith. So if you want healing, stop ART.’ (Focus group 2, female, 33 years)

‘Prayers and anointing oil can heal you. I had a sister – she took ART for 9 months and she stopped. She is just okay up to now - she is not taking them.’ (Participant 5, female, 37 years)

#### Family and partner support and disclosure of HIV status

Some respondents who disclosed their HIV status to their families reported easy adherence because of the support that they received from spouses and children:

‘I’ve disclosed to my family both my wife’s and mine. This disclosure makes it easy for me to take my medication. We remind each other.’ (Participant 7, male, 45 years)

‘If you’re not open to your friends and family and they don’t know, you won’t take your medicine. If you hide you won’t be free. You will wait for people to sleep and then take you medicine (all laugh). By this time your timing for medicine would be past.’ (Focus group 2, female, 28 years)

Other participants, however, reported discrimination from their spouses upon disclosure of their HIV status. This made it difficult for them to openly take their medication:

‘After disclosing I was still troubled. When taking medication, I was hiding and had to be in a private place. My wife was so annoyed, and she started blaming me that I was the one who will cause her to get sick. We had serious problems and our marriage almost ended.’ (Participant 2, male, 34 years)

## Discussion

In our study, many participants reported social barriers to ART adherence in the form of lack of food, which was brought about by high levels of poverty and unemployment. The strong interaction between having adequate nutrition and the taking of medication was illustrated in practical terms when participants chose not to take their ART drugs when food was scarce out of fear of severe side effects. This finding is supported by studies in Uganda and Namibia, in settings where HIV patients were also poor and mostly unemployed.^8,9^ The lack of money also presents a barrier to access the health facility to pick up medication, as confirmed by Ankomah et al. in Ghana.^8^ These findings suggest that structural interventions are necessary to redress poverty among people living with HIV, with specific emphasis on creating income-generating opportunities for them. In addition, health services should consider alternatives to reduce the costs of having patients travel to health facilities to pick up medication, by establishing community pick-up points closer to the patients (in the community).^11^

This study showed that traditional beliefs and practices had strong influences on people’s ability to adhere to ART. This finding is consistent with that of Peltzer et al. which suggests that the use of alternative medicines was gaining popularity globally and may influence more patients to be non-adherent to ART in the future.^10^ Health education to patients should include an awareness of the interactions that traditional medicines may have on patients on ART, and health workers should be sensitised to include this in adherence support and counselling.^12^

Religion in many different forms appeared to play similar roles as traditional medicine because it was also shown in the study to be either used in combination with ART or as a replacement. This is consistent with the findings by Sanjobo et al. in Zambia and Nam et al. in Botswana who have suggested that religion can either facilitate or disrupt ART adherence in various ways.^15,16^ It is imperative that adherence support and counselling should be conducted in a manner that is sensitive to the religious beliefs of ART patients, to counter ill-informed advice as described by Beach et al.^22^

This study found that those who had disclosed their HIV status to their families and close acquaintances had no problems with adherence to ART because of the support rendered, while those who had not disclosed this had challenges regarding adherence to ART. This finding is consistent with that of Obgochi who reported that patients in Malawi who had not disclosed their HIV status were more likely to suffer frequent treatment interruptions.^12^ This finding supports the notion that ART patients should be encouraged to find a treatment buddy or supporter to help them with daily reminders and accountability to take their medication, as suggested by Tsega and colleagues in Ethiopia.^24^

### Limitations of the study

This was an exploratory qualitative study and cannot be generalised to all patients attending LGH. However, it is conceivable that the findings of this study may be applicable to similar resource-constraint settings in other parts of Zambia and the continent. Conducting FGDs and semi-structured interviews within the hospital premises may have inhibited some respondents from expressing their views freely. Lastly, the researcher, being a man, could have limited the depth to which female participants could have contributed because of cultural barriers.

## Conclusion and recommendations

The current exploratory qualitative study conducted at LGH in Zambia demonstrates that factors influencing adherence to ART are complex and interrelated – combining social and economic aspects of HIV and health. The study gives support to the notion that the social determinants of health that influence HIV patients should receive as much attention as medical interventions. Strategies to improve adherence should extend beyond health services and health care to include the general health and social welfare of HIV patients, particularly the need for adequate nutrition.
